# Concert experiences in virtual reality environments

**DOI:** 10.1007/s10055-023-00814-y

**Published:** 2023-06-05

**Authors:** Kelsey E. Onderdijk, Lies Bouckaert, Edith Van Dyck, Pieter-Jan Maes

**Affiliations:** IPEM, Department of Arts, Music and Theatre Sciences, Ghent University, Ghent, Belgium

**Keywords:** Virtual reality, Music concerts, Livestream, Social connection, Presence, Uniqueness

## Abstract

Spurred by recent advances in digital technologies, virtual concerts have become established modes for event attendance and represent a rapidly growing segment of the music industry. Yet, up to now, general experience of virtual concert attendees remains largely underexplored. Here, we focus on a subcategory in this domain: music concerts in virtual reality (VR). Our approach is situated within the theoretical framework of embodied music cognition and entailed investigation through a survey study. Responses of seventy-four VR concert attendees were collected, consisting of demographics, motivations, experiences, and future perspectives. In contrast to previous research, which generally identified social connectedness as a main motivator for concert attendance, our sample regarded it as one of the least important incentives. On the other hand, in line with previous studies, ‘seeing specific artists perform’ and ‘uniqueness of the experience’, were pivotal. The latter was mostly fueled by the possibility to experience/interact with visuals and environments considered as unconceivable in the real world. Furthermore, 70% of our sample regarded VR concerts as ‘the future of the music industry’, mainly relating to the accessibility of such events. Positive evaluations of VR concert experiences, as well as future perspectives regarding the medium, were significantly influenced by the level of experienced immersivity. To our knowledge, this is the first study to provide such an account.

**Supplementary Information:**

The online version contains supplementary material available at 10.1007/s10055-023-00814-y.

## Introduction

Music consumption patterns have changed fundamentally over the last decade. Increasingly, engagement with music occurs online. People use streaming services such as Spotify to discover new music (Datta et al. 2018; Aguiar and Waldfogel 2018), audiovisual platforms such as YouTube to watch music videos (Cayari 2011; Khan 2017) or social media platforms like TikTok to create and share dance videos to the latest hit songs (Kennedy 2020). From 2019 onwards, this trend was additionally spurred by the COVID-19 pandemic, as virtual engagement was often one of the only means to engage with music while still incorporating (some of) its social facets. A vast number of musicians performed at livestream concerts (Khalid 2020; Ren 2020; Weaver et al. 2020; Vandenberg et al. 2021), and while livestreaming is not an entirely new phenomenon, it has been proposed that the pandemic could have accelerated the shift towards an increasingly virtual music industry (Lee et al. 2020).

Speculations on the future of such a virtually situated industry often include advancements in Virtual Reality (VR) (Charron 2017; Breese et al. 2020; Onderdijk et al. 2021). As a rapidly developing and increasingly accessible set of technologies, VR provides radical novel ways for people to interact with digital information/data and engage in remote (online) social experiences. This gives VR a pivotal role in shaping the future of the internet, contributing to the development of what is now generally coined as ‘the metaverse’ (Radoff 2022; Park and Kim 2022). Although multifaceted and wide in scope, the metaverse can be defined as a collection of (partly) computer-generated 3D virtual environments where users, embodied as avatars, can engage in political, economic, social, and cultural activities (Park and Kim 2022, p. 4211). The music industry explores the metaverse as a space to perform, experience and reimagine music. From a commercial point of view, it offers new ways to monetize music, leading to renewed business models and corresponding financial investments boosting and innovating the music industry. We witness the rise of music metaverse production studios (Xyris, Stage11, Wave XR), as well as music concert experiences on dedicated platforms (e.g., MelodyVR, Sansar, VRTIFY, NOYS VR, AmazeVR, Sensorium Galaxy-PRISM World), integrated in more generic social VR platforms (e.g., AltspaceVR, VRChat, Meta's Horizon, Mozilla Hubs, High Fidelity, Anyland, NeosVR) or in social games (e.g., Fortnite, Minecraft, Roblox). In more experimental realms of art music, new opportunities are explored for sound production and experience (e.g., Patchxr, Ars Electronica-Metaverse). Yet, given the significant financial and creative impulses in VR music concert developments, scientific work on the user experience is largely unexplored.

We believe that more insight into the attitudes, motivations, experiences, and desires of users is of utmost importance for further musical VR developments. The present survey study on immersive VR concert attendance aims to meet this need by examining these aspects and reflecting on the similarities and contrasts with the existing body of work on live (streamed) music events. Since past work suggested that, commonly, our strongest musical experiences take place at live concerts (Lamont 2011) and favored musical experiences involve live music performance (Krause et al. 2020), in this study, we aim to explore how such musical experiences translate to concerts in immersive VR.

We approach our investigation from the theoretical framework of embodied music cognition and interaction (Leman 2007; Lesaffre et al. 2017). Within this framework, the role of coupled action-perception processes is fundamental to human interaction with the (musical) world, as it underpins musical perception and cognition, affect and emotion, reward, and motivation, as well as social interaction. Immersive technologies are interesting from the perspective of embodied music interaction, particularly in relation to coupled action-perception processes. A basic tenet of immersive multisensory displays is their presentation of computer-generated stimuli from the perspective of the user (cf. first-person, 1P perspective). For that purpose, immersive display technologies are complemented with bodily interfaces to adapt multisensory stimuli in response to movements and actions of the user’s head and body, in an increasingly direct and transparent (apparent non-mediated) way (Lombard and Ditton 1997). In that regard, it can be argued that immersive technologies intervene in coupled action-perception mechanisms similar as those involved in human interaction with the physical, non-computer-mediated world, and contribute fundamentally to *digital embodiment* (Beaufils and Berland 2022). In this realm of research, it is particularly interesting to investigate the impact of immersive technologies on augmented forms of music interaction and experience, such as those related to the feeling of presence or social connectedness.

To obtain a better grasp of embodied aspects of VR music concert attendance, we surveyed various components of attendees’ experiences and motivations via a mixed-method approach combining qualitative and (validated) quantitative measures. As research on user experiences of VR concerts is only burgeoning, we favored a general, exploratory, and multi-faceted approach that could set the future path to more in-depth, focused, and controlled research. Knowledge about user experiences in musical VR is important for innovating rapidly emerging practical developments in a domain which has a transformative potential for the cultural and creative sector. In addition, the disruptive nature of VR-mediated experience and interaction may contribute substantially to empirical musicology, not only by introducing new methodologies to study embodied music interaction (Van Kerrebroeck et al. 2021), but also by possibly bringing in unexpected observations and findings that go unnoticed in traditional music experience and interaction.

An important part of the survey study focused on social aspects of the VR concert experience. For that purpose, we relied on methods by Brown and Knox (2017), who investigated motivations behind live music attendance and defined four key themes: *Experience*, *Engagement*, *Novelty*, and *Practical.* In our study, these were applied to examine the motivations of VR concert attendees and were supplemented with an additional category focusing on *VR related* motivations (see *Methods* for specifics). Focusing on live concerts, Brown and Knox (2017) revealed that ‘experienced feelings of togetherness’ were generally marked as decisive grounds for attendance. Others showed that ‘being able to share the experience with like-minded others’, as well as ‘interacting with performers’, were commonly considered to be key motives for attending live music events as well (see e.g., Holt 2010; Pitts 2014; Radbourne et al. 2014; Brown and Knox 2017; Tarumi et al. 2017; Krause et al. 2020). Interestingly, social grounds also proved to be crucial for livestreamed event engagement. Extending the scope of music concerts, multiple studies stressed the relevance of social interaction and sense of community for a satisfying livestream experience (Brandtzæg and Heim 2009; Hamilton et al. 2014; Friedländer 2017; Hilvert-Bruce et al. 2018; Skjuve and Brandtzaeg 2019). Although research specifically targeting livestreamed concerts is scarce, the available examples demonstrate that social variables are highly valued here as well, albeit in different forms. Nguyen (2018) found that attendance of livestreamed classical concerts contributed to the overall sense of community, as well as the social and musical experience. In this context, at least to some extent, livestreaming helped to break out of rigid conventions of classical music culture, opening new possibilities to interact with others and express appreciation throughout the course of the concert. Similarly, when compared to pre-recorded music concerts, Swarbrick et al. (2021) uncovered associations between livestreamed concerts and increased feelings of social connectedness, while in a series of experimentally controlled livestream concerts, Onderdijk et al. (2021) emphasized the ability of VR livestreams to facilitate social connectedness, extending that of traditional livestreamed concerts (e.g., using a computer or TV screen). However, evidence also indicates that such effects might depend on certain characteristics of the event. Focusing on livestream rave party attendance during the first months of the COVID-19 pandemic, Vandenberg et al. (2021) demonstrated that such events evoked (some degree of) social frustration in most of the participants. As one of the key features of rave parties is to dance together with others, which is inhibited in its online variant, it could be suggested that livestreams might not prove to be adequate alternatives for all types of music events, at least with regards to their social properties.

Adding to our focus on social aspects of VR concerts, we also stressed the significance of the concept of *presence* during livestream (VR) concert attendance (cf. Onderdijk et al. 2021; Swarbrick et al. 2021). Presence is generally defined as ‘the subjective experience of being in one place or environment, even when one is physically situated in another’ (Witmer and Singer 1998). In other words, it relates to a subjective evaluation of being present in a digital environment, in which the awareness of a mediating technology has dissolved (Lombard and Ditton 1997; Slater and Wilbur 1997; Tamborini and Bowman 2010; Smolentsev et al. 2017; Pallavicini 2019). Although often used interchangeably, it differs from immersion in the sense that presence relates to a psychological experience, while immersion refers to the technical capability to generate experiences in a realistic manner, removing people from their physical reality (Slater and Wilbur 1997; Oh et al. 2018). In the present study, we distinguish between highly and minimally immersive environments, where the former refers to the use of VR headsets and the latter to a 360-degree concert view monitored through a screen. As stated, presence is of interest in the context of music concerts in VR, as it is believed to enhance feelings of social connectedness in digital environments (Durlach and Slater 2000; Ijsselsteijn et al. 2003; de Kort et al. 2007; Slater 2009; Kang and Gratch 2014; Onderdijk et al. 2021; Caldas et al. 2022). Hence, in line with previous studies by for instance Brown and Knox (2017) and Onderdijk et al. (2021), we hypothesized that social connectedness would serve as a strong attendance motivator at music concerts in VR and that the level of immersivity would impact the degree of experienced social connectedness.

Another pivotal motivator for live concert attendance is *uniqueness*. Studies have shown that, in general, individuals like to be part of something unique, that is, to watch artists perform unreleased or rarely performed songs, as well as varied renditions of recorded songs in a live setting and to be able to boast about having attended at a specific time and place (Black et al. 2007; Brown and Knox 2017). Previous research examining distinctions between live (i.e., real-time) and pre-recorded concerts emphasized the relevance of temporal co-presence (i.e., ‘being there’ with others in real-time) for general appreciation and engagement (Shoda and Adachi 2015; Shoda et al. 2016; Swarbrick et al. 2019), although some did not experience discrepancies between the two modes (Belfi et al. 2021). Yet it should be noted that the idea of what constitutes a ‘live’ performance remains under debate and evolves constantly (e.g., Phelan 1993; Auslander 2008). Here, we define livestream concerts as musical performances streamed over the internet at a specific time and place and thus this definition includes the temporal co-presence of attendees. While the notion of ‘being there’ at a specific place has generally been a concern for more traditional livestreams, enhancement of the sense of presence through streaming in VR might, at least to some extent, resolve this issue (Charron 2017). As such, we hypothesized that (highly immersive) VR concerts would be able to provide a sense of participation at a unique time and (virtual) place and as such contribute to the uniqueness of the experience, embodying an essential motivator for concert attendance in VR.

In addition to motivational aspects, we also examined satisfaction with the concert experience, comparing VR with in-person/real-life concert contexts. Highly immersive environments were expected to positively interact with experience, and we presumed such settings to increase overall sense of ‘being there’. Lastly, to provide more insight into this rapidly changing industry from the viewpoint of the user, we inquired about attendees’ future perspectives. In a large-scale study by Bandsintown, over 80% of artists reported they were willing to make live-streaming a permanent part of their performance plans even after in-person concerts are resumed, while more than 60% of music listeners expressed the intention to continue livestream concert engagement after music venues are reopened (Götting 2021a, b). Thus, virtual alternatives are generally believed to become/remain established modes for music concert attendance. To our knowledge, this is the first study to explore the experiences and attitudes of VR concert attendees in this manner and thus contributes to the existing body of work on concert experiences.

## Methods

### Data collection

Data were collected through an anonymous online survey using Google Forms, which was distributed from January 26 to March 14, 2021. The survey was advertised through a wide range of online channels (i.e., VR communities on Reddit, Facebook, Twitch, Discord, and websites of VR developers), inviting individuals with previous experience of VR concert attendance (with different degrees of immersivity) to participate. For the online social media platforms (Reddit, Facebook, and Discord), we targeted subreddits, groups, and communities with a specific link to VR and/or music (e.g., r/virtualreality, r/youtube360, Discord/Virtual Reality, Facebook/VR raves and concerts; see Supplementary Material 1 for a full list). The survey could be filled out in Dutch or English and informed consent was asked at the start of the survey. No financial incentive for participation was provided. All procedures were approved by the ethical committee of the authors’ institution.

### Measures

To obtain a broad view on the embodied and motivational aspects of VR concert experiences, we opted for a mixed-method approach, combining a qualitative and quantitative assessment of user habits and experiences. The qualitative segment consisted of open-ended questions, while the quantitative segment used multiple-choice questions and Likert scale responses, based on existing validated measurement scales, complemented with custom-developed questions. Questions can be categorized in six main sections (for the full survey, see Supplementary Material 2):

1. *General Background (Q1–10)*: This section ascertained demographics as well as musical background and behavior (e.g., musical education, listening habits, concert attendance before COVID-19 restrictions, etc.).
2. *Technologies and Contexts (Q11–19)*: In this part, we surveyed the employed technologies and equipment (e.g., screens, VR headset, audio equipment), concert specifics (e.g., music genre, attendance with first- or third-person view), and contextual features (e.g., being part of specific VR communities, platform usage, how respondents heard about concerts).
3. *Motivations (Q20–21):* This section was modelled to work by Brown and Knox (2017) where motivations to attend (physical) ticketed concerts were examined. Four key themes were identified: *Experience*, *Engagement*, *Novelty,* and *Practical*. *Experience* consisted of witnessing visual effects during a performance (e.g., light shows), the artist(s) and the possibility to be part of something unique. *Engagement* comprised of sharing the experience with other people in the audience, feeling togetherness with other people in the audience, meeting people from all over the world and feeling togetherness with the artist(s). *Novelty* was questioned by whether they were motivated to discover new music. *Practical* consisted of the possibility to stay at home (as they reasoned individuals can listen to ‘perfect recordings’ at home) and ticket price. In the current study these items were supplemented with specific *VR related* motivations, consisting of the possibility to (re)watch the concert at a moment of one’s own choice, having a better view (than one would have at a physical concert), not having to be quiet during the concert, and the ability to stop watching at any time and the possibility to change places/view during the concert. All items were assessed using Likert scales ranging from 1 (no reason for attendance) to 7 (major reason for attendance).
4. *Concert Experience (Q22–51)*: To assess VR concert experience, social involvement and feelings of presence were surveyed. For this, we inquired about the experience of social connectedness with artist(s) and audience. Additionally, a selection of statements from the Igroup Presence Questionnaire (IPQ) was used to assess respondents’ feelings of presence (Schubert et al. 2001). The IPQ consists of a scale for assessing the sense of presence experienced in a virtual environment and aims to measure three components, i.e., spatial presence, involvement, and experienced realism.
5. *Comparison Physical Attendance (Q52–58)*: This section ascertained the attitudes and opinions of respondents regarding perceived (dis)advantages of VR compared to physical concert attendance. It comprised seven Likert scales and four open-ended questions, all based on the VR perspective (e.g., “Are VR concerts more or less accessible than physical concerts?”).
6. *Personal View and Future Outlook (Q59–60)*: This part ascertained personal views on the future of the VR concert scene and included four Likert scales.

Finally, respondents had the opportunity to leave additional comments before submitting the survey.

### Data analysis

Data were processed in Microsoft Excel. R version 4.1.1 (R Core Team 2022) was used for data analysis. All functions used were part of the base R environment unless stated otherwise.

## Results

### Respondents

Seventy-four valid responses were collected. Respondents’ ages ranged from 18 to 72 years (*M* = 34.7, SD 11.4), while their musical experience varied between 0 and 40 years. Those with musical experience had an average of 18.2 years of experience (SD 11.2). Regarding VR concert attendance, all but one respondent provided information on the number of previously attended VR concerts, showing a wide variety ranging from 1 up to 100 (*M* = 6.1, SD 12.4). Further information on demographics, musical background and listening habits, physical concert attendance frequency, and tendency to miss attending such concerts is provided in Table 1.

**Table 1:** Respondents

	*n*	%
*Gender*		
Man	60	81
Woman	14	19
*Country of residence*		
USA	19	26
Belgium	17	23
UK	13	18
Germany	8	11
The Netherlands	4	5
Other	13	18
*Have you engaged in musical training (incl. autodidact)?*		
Yes	43	58
No	31	42
*Daily music listening habits*		
Less than one hour	4	5
One to two hours	30	41
Three to four hours	22	30
Five to eight hours	15	20
Nine hours or more	3	4
*Physical concert attendance before COVID-19*		
Hardly ever	10	14
Less than once a month	44	59
Once or twice a month	17	23
Once a week	2	3
More than once a week	1	1
*How much do you miss attending concerts while being physically present?*		
Not at all to slightly	16	22
Neutral	7	9
Moderately to extremely	51	69

### Technologies and contexts

Fifty-nine percent of respondents (*n* = 44) indicated that they used a VR headset. Most used headsets were manufactured by Oculus (70%), followed by HTC (11%), Sony (9%), Valve (7%), and Samsung (5%). Seventy-eight percent (*n* = 58) indicated that they used a first-person perspective (i.e., “through the eyes of my own character/avatar”), while 31% (*n* = 23) used a third-person perspective (i.e., “behind/above my character/avatar”) during VR concerts. When asked how they learned about these events, 59% (*n* = 44) indicated that they were notified through social media, 24% (*n* = 18) through family and/or friends, 15% (*n* = 11) through online games, and 26% (*n* = 19) by means of a variety of other channels (e.g., VR websites, newsletters, advertisements). Seventy respondents specified the employed VR platforms, with 16% (*n* = 11) using VR Chat, 21% (*n* = 15) YouTube, 11% (*n* = 8) Melody VR, 11% (*n* = 8) Oculus Venues, and 7% (*n* = 5) Wave VR. Some platforms were used by only one or two respondents, such as Altspace VR, Soundstorm, Fortnite, and Minecraft. Thirty-four percent of all respondents (*n* = 25) disclosed to be part of a particular VR community (e.g., AltSpace VR, Loner, VR Chat, Oculus, Reddit, Discord, Tomorrowland). The music genres (*n* = 73) played at the attended concerts are displayed in Table 2.

**Table 2:** Music genres

	*n*	%
Pop	33	45
Techno	29	40
Dance	28	38
Rock	28	38
Classical	12	16
Folk	7	10
Jazz	7	10
Electronic	6	8
Metal	3	4
Other (e.g., hiphop, punk, psytrance, …)	12	16

**Fig. 1:**
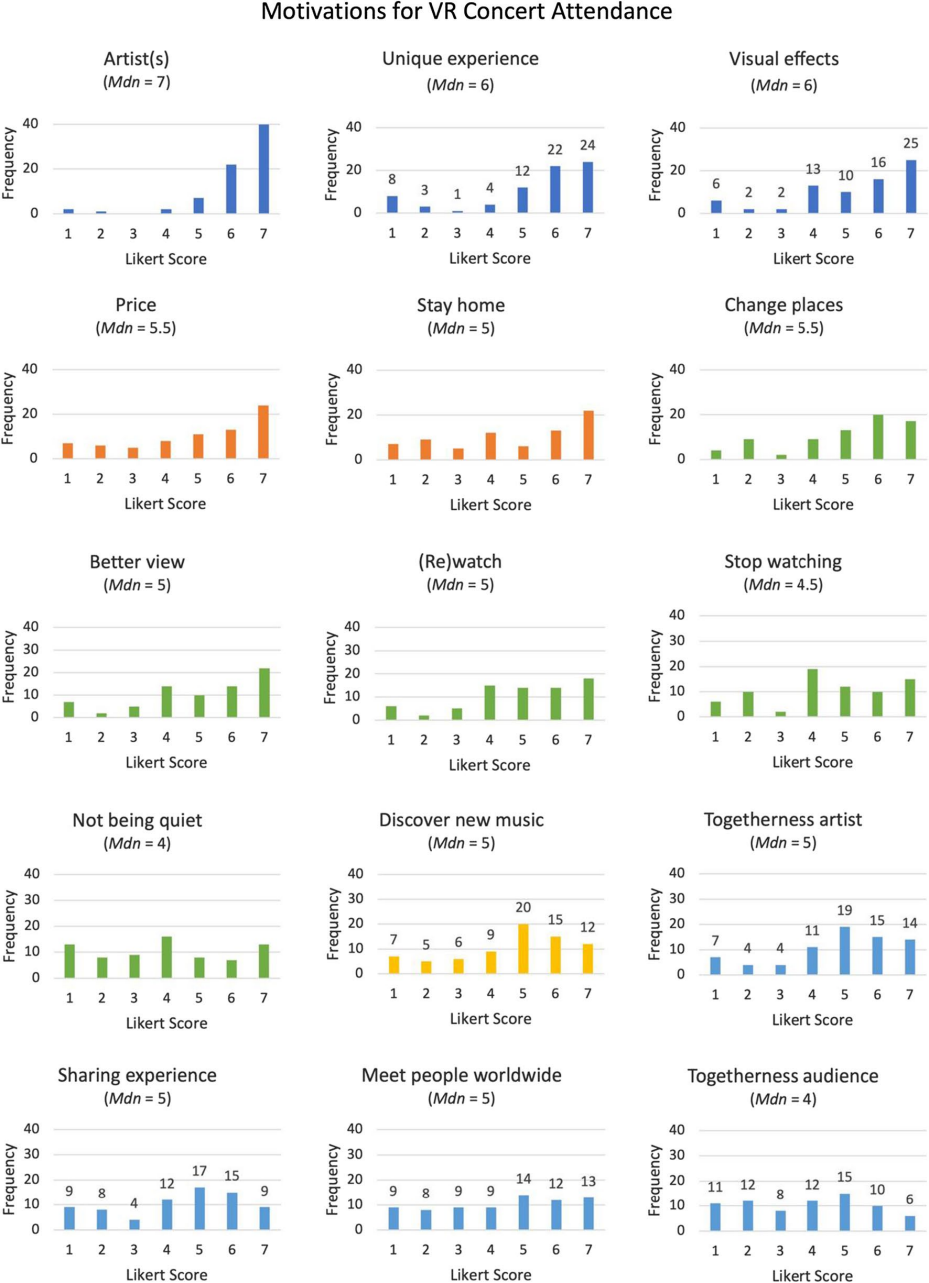
Likert score response distributions for motivations to attend VR concerts

### Motivations

Using Likert scales, respondents rated a set of items evaluating their motives to attend VR concerts (see Fig. 1). The highest scores were obtained for the *Experience* category, exemplifying the importance of the artist(s), uniqueness of the experience, as well as the visual effects. In the open questions on motivation, respondents (*n* = 49) explained (gender and age are provided):

*I’d watch all my favorite artists live in concert on any platform. It’s all about the artists’ pull to watch them live, physically or virtually*. (M, 41)

*I enjoy seeing the stage sets and lighting. I also really like being able to watch the musicians play their instruments up close*. (M, 51)

This was followed by the *Practical* category, which included concert fees and the possibility to stay at home. To open questions assessing motivation, respondents reported benefits related to reduced transport times and distances, decreased expenses, as well as improved accessibility for individuals with physical, social, and/or mental disabilities. The latter is demonstrated by the following statement:

*I can do it from my room, in my house. I suffer from severe social anxiety and PTSD, so I cannot attend concerts live. I can barely leave my house, actually. So that is (a) way for me to have a kind of comfortable concert experience*. (M, 25)

The third most important category was *VR related*. Being able to easily change viewpoints, having better views (compared to real-life concerts), and being able to (re)watch the concert at any given time were key motives in this category. The ability to leave at any moment or not having to be quiet during the concert were regarded as somewhat less important. The open-ended questions on *VR related* motivations did not provide further insights into these categories.

Although *Novelty* mainly focused on the discovery of new music, results of the open-ended questions did suggest a potentially broader interpretation of the category in the context of VR concerts, since some expressed being motivated by the experience of something new overall. One respondent explained his motivation for VR concert attendance as follows:

*Experimentation. VR offers possibilities not possible elsewhere*. (M, 72).

The final category was that of *Engagement*. Within this category, the most prevalent motivator was that of togetherness with the artist(s), while togetherness with the audience obtained the lowest score overall. Sharing the experience and meeting people worldwide also received rather humble ratings, yet still scored positively. Responses to open questions included statements that fit the category of *Engagement*, such as supporting the performer and sharing the experience with (long distance) friends. The latter is exemplified by the following statement:

*Occasionally creating shared experiences with (…) friends who live abroad*. (M, 38)

Furthermore, open-ended questions revealed respondents’ motivations to attend VR concerts due to COVID-19-related restrictions, exemplified by the following two statements:

*To compensate for the lack of physical concerts*. (W, 24).

*The only possibilities are online now. Otherwise, I wouldn’t participate, but I want to support the artists*. (M, 35)

Using Kendall rank correlation tests, we explored whether motivations were correlated with frequency of physically attending concerts (prior to the COVID-19 pandemic) and experience of missing physical concert attendance. Multiple comparison correction was performed using Bonferroni adjustments (adjusted *p* values are reported). Moderate negative correlations were revealed for the ability to remain at home with frequency of physically attending (*r*_*τ*_ = − 0.422, *p* < 0.001), as well as with the experience of missing physical concerts (*r*_*τ*_ = − 0.409, *p* < 0.001), meaning that those who attended and missed physical concerts more, were less motivated by the opportunity to attend VR concerts from home.

### Concert experience

The overall VR concert experience was examined, focusing on concepts of (virtual) togetherness and presence. One aspect pertained to the relationship with the artist(s). Most respondents (57%, *n* = 42) experienced a sense of connection with the artist (i.e., scoring 5–7), while 28% (*n* = 21) identified that such a connection occurred only rarely or never (i.e., scoring 1–3). Fifty-four percent (*n* = 40) reported feeling a shared experience with the artist (i.e., scoring 5–7), while 28% (*n* = 21) hardly perceived a sense of shared experience or never did so (i.e., scoring 1–3). Again, the opportunity was given to elaborate through open-ended questions (*n* = 66). Thirty-six percent (*n* = 24) reported that they felt connected with the artist(s) through interaction, illustrated by the following statements:

*It's elusive; I somehow need to know that the artist is constantly reacting to our mood and cues and that this is truly coming alive in the present and not something that could have been pre-recorded*. (M, 38)

*The artist playing towards the camera, which feels like they are just performing for you*. (M, 53)

Similarly, the connection with other audience members was examined. Forty-six percent (*n* = 34) indicated that they rarely or never experienced feelings of togetherness with the rest of the audience (i.e., scoring 1–3), while 39% (*n* = 29) suggested that they often (or even always) have such experiences (i.e., scoring 5–7). Responses to open-ended questions (*n* = 57) showed that this connection was often made explicit through talking/chatting (39%, *n* = 22) or dancing (26%, *n* = 15). One respondent stated:

*You can jump with others and dance. I know it’s not real-life, but it brings me joy to see it. There’s a certain humor to it as well*. (M, 42)

Looking more closely at these inter-audience interactions, 50% (*n* = 37) reported that they rarely or never interact (i.e., scoring 1–3), while 38% (*n* = 28) interacted often or always (i.e., scoring 5–7). In the open-ended questions, most respondents (*n* = 72) explained why they did or did not interact. Those who did interact generally claimed it to be more fun (15%, *n* = 11) and to enhance the experience (13%, *n* = 9). Those who did not, reported that they wished to prioritize the music over social interaction (18%, *n* = 13) or simply had no need for it (14%, *n* = 10). An illustration is provided through the following two statements:

*It takes me out of my concentration. At a live concert, I enjoy myself in the moment without a phone or internet. I try to do that at home too*. (M, 43)

*Because a concert is more than just music, otherwise I just listen to a recording*. (W, 35)

### Presence

To examine presence, a single mean score of the eight statements relating to presence was calculated (see questions 40, 43, 44, 46, 47 and 49 in Supplementary Material 2). Using the *ltm* package (Rizopoulos 2006), Cronbach’s alpha was calculated, which showed acceptable reliability of the score (*α* = 0.839) (Bonnet and Wright 2014). This provided an average score of 4.601 (SD 1.262). Further insights into the impact of certain facets of this measure (i.e., concentration levels and awareness of the real world) on the VR concert experience were obtained via open questions. Seventy-one respondents reported on aspects in the real world that attracted attention during VR concert attendance, thus hindering presence, such as other people in the room (35%, *n* = 25), auditory elements (e.g., noises; 25%, *n* = 18), and tactile ones (e.g., VR headset and controllers; 17%, *n* = 12). Others mentioned aspects that were beneficial for the level of concentration (*n* = 70), such as (good) technical quality (30%, *n* = 21), the music itself (19%, *n* = 13) and interaction with the audience and artist(s) (16%, *n* = 11).

Additionally, we expected feelings of presence to differ between those who attended with and without a headset. A significant difference between headset users and non-users was indeed retrieved for presence. As we calculated a mean score for this measure, a *t*-test was used. A Shapiro–Wilk test showed no significant deviation from normality, *W* = 0.979*, p* = 0.241, while a Levene’s test, using the *car* package (Fox and Weisberg 2019), indicated equal variances, *F*(1) = 0.006, *p* = 0.940. Results revealed higher presence ratings for those using a headset (*M* = 5.077, SD 1.144) than those who did not (*M* = 3.904, SD 1.107), *t*(72*)* = − 4.386, *p* < 0.001.

Moreover, significant distinctions were found relating to headset use. Higher ratings for shared experience with the artist(s) were retrieved for headset users (*Mdn* = 6, SD 1.785) compared to users without headset (*Mdn* = 4, SD 1.938), *W* = 452, *p* = 0.021. In addition, those using headsets experienced stronger connections with the artist(s) (*Mdn* = 5, SD 1.669) compared to other users (*Mdn* = 4, SD 1.884), *W* = 480.5, *p* = 0.045.

As previous research revealed gender effects regarding experience of presence (Felnhofer et al. 2012), as well as social connection in virtual concerts (Onderdijk et al. 2021), we also examined the role of gender. A Levene’s Test indicated equal variances for this variable, *F*(1) = 0.643, *p* = 0.425. The subsequent *t*-test did not reveal any differences based on gender for the experience of presence, *t*(72) = − 1.370, *p* = 0.175. Yet, a significant difference between men (*Mdn* = 5, SD 1.870) and women (*Mdn* = 3.5, SD 1.785) was found in sharing the experience with the artist(s) using a Wilcoxon rank sum test, *W* = 257, *p* = 0.023.

Lastly, we tested whether presence correlated with frequency of physical concert attendance (prior to the COVID-19 pandemic) and missing the experience of attending these events. Correlation analyses focusing on frequency of physical concert attendance revealed a significant negative correlation with presence, *r*(72) = − 0.253, *p* = 0.029 (computing Pearson correlation coefficient). Yet, no significant correlation was found between presence and missing the opportunity to attend these events, *r*(72) = − 0.145, *p* = 0.217.

### Comparison physical attendance

Most respondents (82%, *n* = 61) considered VR concerts to be more accessible than concerts attended in person (i.e., scoring 5–7), with 47% (*n* = 35) regarding them as far more accessible (i.e., score of 7), and only 12% (*n* = 9) deeming them as less accessible (i.e., scoring 1–3). Responses to the open-ended questions (*n* = 69) specified several aspects facilitating accessibility, such as absence of location-related restrictions (45%, *n* = 31), increased accessibility for individuals with disabilities (13%, *n* = 9), convenience of usage and access (30%, *n* = 21), and price (25%, *n* = 17). Respondents experiencing VR concerts as less accessible mostly related this to (demanding) technological requirements (14%, *n* = 10).

Most respondents (78%, *n* = 58) acknowledged that VR concerts can display visual effects unobtainable at regular concerts. Similarly, for 76% (*n* = 56) the main appeal of VR concerts lies in the fact that they can facilitate and enhance experiences unattainable in real life. Correspondingly, 47% (*n* = 35) deemed VR concerts as more unique (i.e., scoring 5–7), while 30% (*n* = 22) regarded them as less unique (i.e., scoring 1–3), when compared to traditional ones. Some explanation for assessing VR concerts as more unique was provided in the open-ended questions (*n* = 70), which sometimes related to the more ‘spectacular’ properties of such events (16%, *n* = 11). An example:

*Access to normally inaccessible areas of the stage. Being able to concentrate on a different aspect of the concert each time. I've watched some concerts (e.g., Awolnation) 50+ times and I am guaranteed to see new things with each viewing*. (M, 45)

For those considering VR concerts as less unique, some referred to the interaction, which they found flawed (10%, *n* = 7). However, it should be noted that 16% (*n* = 11) explicitly stated to find these two concert modalities rather incomparable, which is exemplified by the following statement:

*Every experience is unique - physically or virtually. One is enjoying from different angles and as long as these services exist and have the opportunity pandemic or not that’s what counts*. (M, 41)

The remaining three Likert scale questions showed that 46% (*n* = 34) agreed with 'being more oneself' during VR concerts, while 34% disagreed (*n* = 25). Yet, 47% (*n* = 35) found it harder to connect with other audience members using VR, while 41% (*n* = 30) had opposite beliefs. In addition, 59% (*n* = 44) of the sample did not experience similar levels of fulfilment at VR concerts compared to physical ones, while 28% (*n* = 21) did report to obtain similar experiences. Two open-ended questions further examined respondents’ views on the added value of VR concerts (*n* = 72), as well as on what they believed might still be lacking (*n* = 74). For both questions, a top five was created consisting of the most popular replies. Thirty-two percent of the responses on added value referred to accessibility (e.g., absence of geographical limits), 24% to the view (e.g., more intimate view, multiple angles, close-ups), 21% to convenience (e.g., absence of pushing crowds, comfortable), 19% to concert fees (e.g., cheaper ticket prices, no travel costs, no expensive food or drinks), and 15% to spectacle (e.g., visual effects, impossible actions and environments). Fifty percent of the responses referring to elements that were perceived to be lacking concerned social experience (e.g., interaction, seeing other people and the artist(s) in real life, singing with the crowd), 41% physical experience (e.g., sensory information, feeling other people), 16% atmosphere (e.g., energy of the crowd), 15% facilities (e.g., food and drinks, merchandise), and 14% technical aspects (e.g., proper technological quality).

Again, to better understand these results, we checked whether these elements varied between minimally or highly immersive contexts and whether ratings differed for individuals regularly attending traditional concerts or those stating to miss attending such events more than others. Some of these factors indeed seemed to have had an impact, e.g., overall, the VR concert experience was more positively assessed in highly immersive contexts, by regular concert attendees and those who missed attending more (see Table 3 for an overview of the results).

**Table 3:** Differences between and influences on VR concert perspectives

Item	Wilcoxon comparisons				Kendall correlations			
	Headset usage				Freq. physical attendance		Missing concerts	
	Minimally immersive	Highly immersive	*W*	*p*	*r*_*τ*_	*p*	*r*_*τ*_	*p*
VR concerts are more accessible than physical concerts	*Mdn* = 6, SD 1.732	*Mdn* = 6.5, SD 1.597	594.5	.445	− 0.215	.381	− .085	–
VR provides novel dimensions	*Mdn* = 5.5, SD 2.097	*Mdn* = 7, SD 1.275	435	.009	− 0.304	**.028**	− .132	–
VR provides more impossible experiences	*Mdn* = 5, SD 2.047	*Mdn* = 7, SD 1.322	389.5	**.001**	− 0.333	**.011**	− .304	**.018**
VR is more unique	*Mdn* = 4, SD 1.754	*Mdn* = 5, SD 1.867	356.5	** < .001**	− 0.198	.464	− .236	.120
In VR, you can be yourself more	*Mdn* = 4, SD 1.868	*Mdn* = 5, SD 1.911	402	**.004**	− 0.383	**.001**	− .407	** < .001**
In VR, it is easier to connect with others	*Mdn* = 2.5, SD 1.768	*Mdn* = 4.5, SD 2.139	412.5	**.006**	− 0.297	**.025**	− .302	**.012**
VR provides the same fulfillment	*Mdn* = 1, SD 1.752	*Mdn* = 3, SD 1.999	397	**.003**	− 0.239	.161	− .318	.007
VR is the future of the music scene	*Mdn* = 6, SD 2.554	*Mdn* = 6, SD 1.599	494.5	.060	− 0.369	.002	− .201	.373
I would attend more VR concerts if possible	*Mdn* = 2.5, SD 2.135	*Mdn* = 6, SD 1.823	269.5	** < .001**	− 0.426	** < .001**	− .460	** < .001**
I prefer VR over physical concerts	*Mdn* = 1, SD 1.906	*Mdn* = 4, SD 2.011	282.5	** < .001**	− 0.421	** < .001**	− .562	** < .001**
I would choose VR concerts even if physical attendance was possible	*Mdn* = 1, SD 1.868	*Mdn* = 4.5, SD 1.954	1067	** < .001**	− 0.379	**.001**	− .484	** < .001**

Results of Wilcoxon comparisons and Kendall correlations. Significance values of correlations are adjusted for multiple (i.e., 11) comparisons using the Bonferroni method

### Future outlook

Seventy percent (*n* = 52) of our sample regarded VR concerts as ‘the future of the music scene’ (i.e., scoring 5–7), while 22% (*n* = 16) did not share this perspective. As, for the time being, the VR concert scene is still rather modest, we inquired whether respondents would engage more often in such events if there were simply more VR concerts organized. A majority (57%, *n* = 42) agreed that they would do so (i.e., scoring 5–7), while 30% (*n* = 22) did not foresee an increased interest (i.e., scoring 1–3). Nevertheless, only 30% (*n* = 22) agreed that they generally preferred VR concerts over physical ones (i.e., scoring 5–7), while half of the respondents (50%; *n* = 37) had opposing beliefs. See Table 3 for an overview (also including headset use as a factor of comparison, as well as Kendall correlations between physical concert attendance prior to the COVID-19 pandemic and feelings of missing physical concert attendance).

## Discussion

Concerts in VR are becoming established events in a rapidly changing music industry. This survey study aimed to enhance our understanding of VR music concert attendance, the contexts in which these events take place, individuals’ motives to attend, as well as the features which facilitate (or hinder) the overall experience. Results indicated that, even prior to the COVID-19 pandemic, our sample of VR concert attendees rarely attended conventional, offline music concerts. This suggests that, compared to the more traditional music scene, VR concerts might attract a rather different population. Moreover, those who attended fewer concerts in person obtained more satisfying experiences in VR compared to the more frequent concert goer. This might imply a different overall stance of these two subgroups regarding their interpretation of a satisfying concert experience, as well as their beliefs regarding the necessary prerequisites to obtain such an experience. For instance, although most previous research identified togetherness as an elementary motive to attend music concerts (Pitts 2014; Radbourne et al. 2014; Brown and Knox 2017; Tarumi et al. 2017; Krause et al. 2020), such feelings only served as moderate motivators for our sample of VR concert attendees. Nevertheless, 69% of our respondents did indicate to (moderately) miss physical concert attendance. Such findings seem to suggest that something might be lacking in the VR experience and thus raises the question on what this might be. Some did indeed indicate that they wished to interact with other audience members, and thus seek social anchoring, which suggests that some caution is needed when interpreting findings regarding motives; possibly, concertgoers might not consider social aspects as pivotal grounds for participation in VR events, not because they do not value such aspects, but rather since they already anticipate the experience to be less ‘communal’ than in real life. Thus, some level of expectancy might be at play here, leveling out particular motivational aspects.

The most vital motive for VR concert attendance was to see (an) artist(s) perform. Multiple response categories in this study (e.g., togetherness with the artist, sharing the experience with the artist, artist as motivator to attend) stressed the relevance of the performer-audience relation. This is in line with previous findings which highlight the emotional and cultural support experienced during performances through expressions of fanhood (Earl 2001; Pitts 2014; Brown and Knox 2017; Swarbrick et al. 2021). In the present study, attendees wanted to see performers up close and aimed to experience real-time interactions with them. Yet, the actual result could still be improved upon, as only a small majority of our study sample stated that they felt (somewhat) connected with the artist(s) during the performance. Thus, a component of the VR concert experience that would benefit from further finetuning is the performer-audience interaction. Our respondents provided some ideas for future improvement, for instance through interacting with (social) cues and moods of the audience or directing the performance towards the camera. Furthermore, alternative forms of communication could also be considered. For example, Wang and Okada (2021) developed an interactive system for livestreaming, providing real-time visual feedback presented through a dynamically burning flame. The intensity of this flame depended on audience members’ heart rates, measured by smart watches. Comparable interactive tools have been developed for live concert settings (e.g., Feldmeier and Paradiso 2007; Yang et al. 2017), which might be worth translating to the virtual realm.

Presumably, such tools might also foster intensified feelings of social connectedness between the audience members themselves. It should be noted, though, that those who indicated that they rarely or never interact with others during concerts suggested that they did not need to interact or reported that they regarded the music to be more important. Previous studies have shown that an increased emphasis on the social components of a virtual environment does not always benefit all. Individuals experiencing (some) discomfort or unease during social interaction, or those who might feel uncomfortable in the presence of others, are likely to be less motivated to increase social interaction and connectedness (Allmendinger 2010; Cortese and Seo 2012; Oh et al. 2018). This can provoke an urge to stay in the background but can also prompt a greater sense of comfort if the social presence of others is minimized (Joinson 2004; Hertel et al. 2008; Hammick and Lee 2014; Oh et al. 2018). Given that most of our respondents did not frequently attend real-life concerts and that a negative correlation was observed between the ability to stay at home and the frequency of attending concerts in real life, our sample might have felt some discomfort related to social interactivity. However, as previously mentioned, respondents did indicate that they missed the social aspects of physically attended live events. Although more research is needed to obtain a better grasp of the matter, to some extent, these aspects could already be considered when developing future (inclusive) virtual environments.

Additionally, in line with Onderdijk et al. (2021), feelings of social connectedness with the artist(s) differed based on gender, with women experiencing less social connection than men. Previously, it has been suggested that such a difference might relate to the feeling of presence (Onderdijk et al. 2021)—which facilitates connectedness (Durlach and Slater 2000; Rettie 2003; Dey and De Giizman 2006; Ijsselsteijn et al. 2003; Kang and Gratch 2014)—as women generally experience less presence in virtual environments than men (Felnhofer et al. 2012). Yet, no such distinction in the experience of presence was found in the current study. Alternatively, to some extent, this finding might be explained through previously observed gender effects on engagement in parasocial interactions (Wang et al. 2008; Onderdijk et al. 2021). However, additional research is needed to understand the role of gender more fully in such contexts.

Furthermore, in line with previous accounts, the uniqueness of the experience was shown to be a key incentive for VR concert attendance. Many referred to the exceptional character of the visual effects, unparalleled in real-life environments, as well as the ability of VR to facilitate experiences that would be impossible to obtain elsewhere. Often, the potential of immersive virtual environments to enable ecologically realistic experiences is put forward (Bailenson et al. 2003), yet it seems that in the case of VR concerts it is mainly the power to achieve ‘the impossible’ that seems to attract and motivate people to attend. To some extent, this might also account for the fact that some explicitly stated that these different concert modes (i.e., physical attendance and virtual attendance) are beyond compare. Correspondingly, Vergauwen (2021) suggested that, rather than endangering one another, future virtual and physical concert spaces will exist alongside each other. In his view, the popularity of virtual concerts will be maintained after the end of the pandemic due to the ability of VR to overcome constraints typically associated with live concert attendance (see e.g., Godbey et al. 2010), such as the opportunity to attend shows (via livestream) even when sold out (thus resulting in hybrid events), the possibility for artists to reach fans located in areas that are not on the tour schedule, or to reduce artists’ as well as audiences’ ecological footprints. Additionally, most of our respondents considered VR concerts to be more accessible. Some acknowledged requirements with respect to technological tools, which might somewhat raise the participation threshold, but overall, increased accessibility as well as reduced charges (e.g., drinks, coat check) were regarded as more persuasive attendance facilitators. In a similar vein, the accessibility for individuals with physical, social, and/or mental disabilities at VR concerts was praised. All in all, these factors might largely explain why 70% of our sample considered VR concerts as ‘the future of the music scene’.

Finally, it is worth mentioning that attitudes towards VR concerts (e.g., wishing to attend more concerts in VR, preferring VR concerts over physical ones) and perceptions of the experience were especially positive for those engaging in highly immersive environments. Sharing the experience and feeling connected with the artist(s) and feeling present were all rated significantly higher by those using VR headsets. The latter indicates that attendees had a greater sense of “being there” when using a VR headset. As such, headset use could potentially resolve issues related to feelings of displacement, which are often experienced at traditional livestreamed concerts (Charron 2017). Intensified feelings of presence, as well as more highly immersive environmental properties, might have facilitated feelings of social connectedness (Durlach and Slater 2000; Ijsselsteijn et al. 2003; Kang and Gratch 2014; Onderdijk et al. 2021).

Positive effects of highly immersive VR on various aspects of the user experience could potentially be explained by the theory of embodied music cognition (Leman 2007; Lesaffre et al. 2017). This theory holds that musical perception, sense-making, and emotion is rooted in active engagement and a direct coupling of human actions with (changes in) the musical environment (e.g., sounds, instruments, musicians, audience). As the highly immersive VR experiences in our study suggest a direct coupling between user (head) movements (using head-mounted displays) and corresponding changes in the perceived musical environments, it may explain the observed effects related to increased feelings of presence (cf. Cummings and Bailenson 2016), social connectedness, and motivation. It should be noted however that increasing immersion does not linearly enhance presence (Oh et al. 2018) and its relation to presence and social connectedness thus warrants further investigation.

### Limitations and future directions

The current study has its limitations. Firstly, as research focusing on immersive virtual concert experiences is still in its infancy (Onderdijk et al. 2021; Slater et al. 2022), rather than using more in-depth qualitative and/or quantitative methods, we opted to take a broad, exploratory view using a mixed-method approach. Our methods provided a variety of novel findings on such experiences, which can be further elaborated on in more in-depth future work.

Also, the sample size warrants prudence. Although this study provides novel findings on VR concert attendance (e.g., used technologies, online community engagement), it should also be noted that many variables remain unknown. One example is the size of concert crowds. Tarumi et al. (2017) pointed out that, to some extent, valuations of concert experiences depend on crowd size (e.g., small versus large festivals). Additionally, livestream research demonstrated that individuals preferring smaller channels (< 500 viewers) were more motivated by social engagement and more inclined to believe that these channels facilitate the most meaningful interactions (Hamilton 2014; Hilvert-Bruce et al. 2018). Similarly, motivations to participate in streaming opportunities were shown to differ between countries/cultures (Friedländer 2017). How these variables translate into music concerts in VR remains a question for future investigation.

Further, we did not examine whether respondents referred to real-time or delayed VR concert attendance. As previous explorations of the concert experience stressed the relevance of temporal co-presence (Shoda and Adachi 2015; Shoda et al. 2016; Swarbrick et al. 2019; Onderdijk et al. 2021), it would be interesting to investigate whether this also applies to VR concerts. Concerts might be streamed live at a certain point in time but attended by a temporally co-present audience at a later moment as well. Alternatively, as exemplified by a quote of one of our respondents, the great number of potential modes (e.g., from different angles each time) for (re)watching a performance might alter how we place value on concert experiences and how we define liveness. Such questions fuel interesting pathways for future research.

Moreover, this study was conducted during the COVID-19 pandemic. Replication will be needed to evaluate whether these findings can be generalized outside of this context. On the other hand, this snapshot in time provides interesting insights into VR concert attendance while it is still in its infancy. Nevertheless, a sudden influx of people who turned to such means to compensate for the absence of real-life events might have resulted in a somewhat atypical sample. Future research could examine closer whether our general findings are typical for people partaking in VR concerts, as well as examine how this relates to the way in which we develop and shape our online communities. Furthermore, our sample includes individuals who had attended VR concerts before, thus represents a group of people who were already open to the idea of attending such concerts. Hence, interpretation of our findings and their implications should be done within this context, while examination of attitudes and future perspectives of the general public might be of interest for future work.

## Conclusion

This study investigated motivations, attitudes, and experiences of VR concert attendees. Our results indicate that the uniqueness of the experience and the relation with the artist(s) are key motivators for attendance. Experiences were positively influenced by headset usage, general accessibility, and the possibility to experience visuals and environments that are unattainable in more conventional settings. However, in contrast to our hypothesis and previous findings, the feeling of togetherness with other audience members only played a minor role as motivator. The development and need for social interaction in VR concerts are of interest for future research. Furthermore, the rather moderate frequency of physical concert attendance in our sample suggests that VR concerts possibly introduce new types of audiences. Altogether, this study provides valuable insights into the current frontiers of virtual concert spaces.

## Supplementary Information

Below is the link to the electronic supplementary material.

Supplementary file1 (DOCX 19 kb)

Supplementary file2 (DOCX 34 kb)

## Data Availability

The datasets generated during and/or analyzed during the current study are available from the corresponding author on reasonable request.
