# Supplementary material for: Concert experiences in virtual reality environments
Source: Virtual Real. 2023 Jun 5:1–14. Online ahead of print. doi: 10.1007/s10055-023-00814-y (PMC10239717; doi:10.1007/s10055-023-00814-y)
Supplement: Supplementary file 1 — Supplementary file1 (DOCX 19 kb) [file 10055_2023_814_MOESM1_ESM.docx]

| Reddit | Discord | Facebook Pages/Groups | Companies/Communities |
| --- | --- | --- | --- |
| r/EDM | Virtual Reality | Virtual Reality | Soundstorm |
| r/Music | VRChat Community | VR Basement Group | Lockdown Escape |
| r/ExtendedReality |  | Oculus Quest Community | VR Base |
| r/virtualreality |  | SteamVR | Poppr |
| r/oculus |  | Sidequest VR | VR Mania |
| r/GearVR |  | Virtual Reality & Augmented Reality | Be Virtual |
| r/SteamVR |  | Oculus Quest 2 Community | Level Up Gent |
| r/virtuality |  | Virtual & Augmented Reality | ASIL |
| r/WeAreTheMusicMakers |  | The Official Virtual Reality-Augmented Reality-Interest-Group | + additional personal connections (names removed for privacy reasons) |
| r/SampleSize |  | VR Raves and Concerts |  |
| r/musicians |  | Virtual Reality Creative Community |  |
| r/AndroidVR |  | Virtual Reality (VR) Gaming Community |  |
| r/redditmusicclub |  | Augmented/Virtual Reality Developers |  |
| r/Gear360 |  | Oculus Quest 2 |  |
| r/VRFilm |  | Oculus Quest Gaming Community |  |
| r/youtube360 |  | Oculus Quest 2 & Rift central |  |
| r/TheWaveVR |  | Oculus Virtual Reality |  |
| r/mixedreality |  | VR Players |  |
| r/GoogleCardboard |  | Oculus Quest 2 |  |
| r/OculusGo |  | Conservatorium Kortrijk |  |
| r/Tomorrowland |  | International Opera Academy |  |
| r/VRPhysics |  | Dé Academie Ieper |  |
| r/NextVR |  | Festival Dranouter |  |
| r/PS4 |  | Muziekschool Ars Musica Gent |  |
| r/OculusQuest |  | VI.BE |  |
| r/OculusriftS |  |  |  |
| r/Techno |  |  |  |
| r/classicalmusic |  |  |  |
| r/indieheads |  |  |  |
| r/futurebeats |  |  |  |
| r/DnB |  |  |  |
| r/Coldplay |  |  |  |
| r/PSVR |  |  |  |
| r/billieeilish |  |  |  |
| r/Twitch |  |  |  |
| r/Takemysurvey |  |  |  |
| r/Tinashe |  |  |  |
| r/jeanmicheljarre |  |  |  |
| r/TheWeeknd |  |  |  |
| r/marshmello |  |  |  |
| r/AlisonWonderland |  |  |  |
| r/travisscott |  |  |  |
| r/Postmalone |  |  |  |
| r/Hololive |  |  |  |
| r/kpop |  |  |  |
| r/Jazz |  |  |  |
| r/Gent |  |  |  |
| r/PS4Pro |  |  |  |
