# Supplementary material for: Concert experiences in virtual reality environments
Source: Virtual Real. 2023 Jun 5:1–14. Online ahead of print. doi: 10.1007/s10055-023-00814-y (PMC10239717; doi:10.1007/s10055-023-00814-y)
Supplement: Supplementary file 2 — Supplementary file2 (DOCX 34 kb) [file 10055_2023_814_MOESM2_ESM.docx]

# Supplementary Material – Survey (English version)

| **Overview of the survey categories**  CATEGORY 1: General background  CATEGORY 2: Technologies and Contexts  CATEGORY 3: Motivations  CATEGORY 4: Concert experience  4.1 Social aspects  4.2 Emotional aspects  4.3 Presence aspects (IPQ)  Component 1: Spatial presence  Component 2: Involvement  Component 3: Experienced realism  CATEGORY 5: Comparison Physical Attendance  CATEGORY 6: Personal View and Future Outlook |
| --- |

**CATEGORY 1: General background**

**Q1.** Do you agree with the statements above? Yes (1) – No (2)

**Q2.** Are you 18 years or older? Yes (1) – No (2)

*<when indicated ‘No’ on one or both questions, the survey was terminated>*

Section 1: Demographics

**Q3.** What is your age? *<integer input>*

**Q4.** What is your gender?

- Female (1)
- Male (2)
- Prefer not to say (3)
- Other: … <*free text input>* (4)

**Q5.** What is your country of residence? <*free text input>*

**Q6.** What kind of musical education do you follow or have you followed? *<multiple answers possible > <coded as 0 = no, 1 = yes>*

- Higher education (e.g., Conservatory)
- University (e.g., Musicology)
- Private courses
- Music academy
- Self-thought (i.e., autodidact)
- None
- Other: ……………… <*free text input>*

**Q7.** How many years have you been playing music? *<integer input>*

**Q8.** On average, how many hours a day do you listen to music? This includes listening to music as main activity as well as background activity.

- 0 hours (1)
- 1-2 hours (2)
- 3-4 hours (3)
- 5-8 hours (4)
- 9 or more (5)

**Q9.** Before the COVID-19 restrictions, how often did you attend concerts (while being physically present)?

- Never (1)
- Less than once a month (2)
- Once or twice a month (3)
- Once a week (4)
- More than once a week (5)

**Q10.** How much do you miss attending concerts while being physically present?

*<7-point Likert scale ranging from ‘not at all’ (1) to ‘very much’ (7)>*

**CATEGORY 2: Technologies and Contexts**

**Q11.** What type of screen did you use to watch concerts in VR? *<multiple answers possible >* *<coded as 0 = no, 1 = yes>*

- (Big) TV screen
- VR headset
- Laptop screen
- Desktop screen
- Large projection
- Phone screen
- Other: ……………… <*free text input>*

**Q12.** If you used a VR headset, what brand is it? <*free text input>*

**Q13**. What type of audio did you use to listen to concerts in VR? *<multiple answers possible > <coded as 0 = no, 1 = yes>*

- Headphones: basic
- Headphones: high quality
- Headphones: noise cancelling
- Speakers: basic
- Speakers: high quality
- Speakers: stereo
- Speakers: surround
- Other: ……………… <*free text input>*

**Q14**. Which platform(s) did you use to watch concerts in VR? <*free text input>*

**Q15**. If applicable, which VR community are you a member of? <*free text input>*

**Q16**. What perspective onto the concert did you use? *<multiple answers possible > <coded as 0 = no, 1 = yes>*

- First-person perspective: “through the eyes of my own character/avatar”
- Third-person perspective: “behind/above my character/avatar”

**Q17**. How did you find out about VR concerts? *<multiple answers possible > <coded as 0 = no, 1 = yes>*

- Social media
- Friends or family
- While playing a game
- Other: ……………… <*free text input>*

**Q18**. How many concerts in VR have you already attended? *<integer input>*

**Q19**. Of which music genres have you already attended a concert in VR? *<multiple answers possible > <coded as 0 = no, 1 = yes>*

- Classical music
- Pop
- Rock
- Folk
- Jazz
- Dance
- Techno
- Other: ……………… <*free text input>*

**CATEGORY 3: Motivations**

**Q20**. Please indicate to what extent the following aspects were reasons for you to attend a concert in VR.

|  | Not at all (1) | Not (2) | Rather not (3) | Neutral (4) | Rather yes (5) | Yes (6) | Very much (7) |
| --- | --- | --- | --- | --- | --- | --- | --- |
| **Experience** | | | | | | | |
| Visual effects |  |  |  |  |  |  |  |
| The artist(s) |  |  |  |  |  |  |  |
| The possibility to be part of something unique |  |  |  |  |  |  |  |
| **Engagement** | | | | | | | |
| Sharing the experience with other people in the audience |  |  |  |  |  |  |  |
| Feeling a togetherness with other people in the audience |  |  |  |  |  |  |  |
| Meet people from all over the world |  |  |  |  |  |  |  |
| Feeling a togetherness with the artists |  |  |  |  |  |  |  |
| **Novelty** | | | | | | | |
| Discovering new music |  |  |  |  |  |  |  |
| **Practical** | | | | | | | |
| You can stay at home |  |  |  |  |  |  |  |
| Concert tickets are cheap or for free |  |  |  |  |  |  |  |
| **VR related** | | | | | | | |
| Possible to (re)watch the concert at a moment of your choice |  |  |  |  |  |  |  |
| A better view |  |  |  |  |  |  |  |
| Don’t have to be quiet for others during the concert |  |  |  |  |  |  |  |
| The ability to stop watching at any time |  |  |  |  |  |  |  |
| The possibility to change places during the concert |  |  |  |  |  |  |  |

**Q21.** Were there any more reasons why you would attend a concert in VR? <*free text input>*

**CATEGORY 4: Concert experience**

**4.1 Social aspects**

**-----------------------**

**Q22.** Do you invite people to watch VR concerts together?

*<7-point Likert scale ranging from ‘never’ (1) to ‘always’ (7)>*

**Q23.** What is the reason you do or do not invite other people? <*free text input>*

**Q24.** When attending the VR concert, did you interact with other people in the virtual audience?

*<7-point Likert scale ranging from ‘never’ (1) to ‘always’ (7)>*

**Q25.** Why is interaction with other people during the concert (not) important for you? <*free text input>*

**Q26.** How important is it for you to feel connected with other people in the audience during a VR concert?

*<7-point Likert scale ranging from ‘not important at all’ (1) to ‘very important’ (7)>*

**Q27.** Based on all the VR concerts you’ve seen, how often did you feel connected to others in the audience?

*<7-point Likert scale ranging from ‘never’ (1) to ‘always’ (7)>*

**Q28.** How did this connection with other people in the audience express itself or not? <*free text input>*

**Q29.** How important is it for you to feel connected to the artist(s) during a VR concert?

*<7-point Likert scale ranging from ‘not important at all’ (1) to ‘very important’ (7)>*

**Q30.** Based on all the VR concerts you’ve seen, how often did you feel connected to the artist(s)?

*<7-point Likert scale ranging from ‘never’ (1) to ‘always’ (7)>*

**Q31.** How did this connection with the artist(s) express itself or not? <*free text input>*

**Q32.** To what extent do you find VR concerts a lonely or social activity?

*<7-point Likert scale ranging from ‘very lonely’ (1) to ‘very social’ (7)>*

**Q33.** Why do you think VR concerts are lonely or social? <*free text input>*

**4.2 Emotional aspects**

**----------------------------**

**Q34.** How important is it for you the feel emotionally involved during a VR concert?

*<7-point Likert scale ranging from ‘not important at all’ (1) to ‘very important’ (7)>*

**Q35.** Based on all the VR concerts you’ve seen, how often did you feel emotionally involved?

*<7-point Likert scale ranging from ‘never’ (1) to ‘always’ (7)>*

**Q36.** What made you feel (not) emotionally involved during the VR concert? <*free text input>*

**Q37.** Describe the best VR concert you’ve seen so far. Please mention which concert it was. <*free text input>*

**Q38.** Describe the worst VR concert you’ve seen. Please mention which concert it was. <*free text input>*

**Q39.** Please indicate to what extent each statement applies to your experience of VR concerts.

|  | Strongly disagree (1) | Disagree (2) | Slightly disagree (3) | Neutral (4) | Slightly agree (5) | Agree (6) | Strongly agree (7) |
| --- | --- | --- | --- | --- | --- | --- | --- |
| I shared emotions with others in the virtual audience during the VR concert. |  |  |  |  |  |  |  |
| I felt like I was sharing the experience with the artists. |  |  |  |  |  |  |  |
| I like attending concerts of artists I don’t know yet. |  |  |  |  |  |  |  |

**4.3 Presence aspects (IPQ)**

**----------------------------------**

**Based on the IPQ and its three main components ‘Spatial presence’, ‘Involvement’, and ‘Experienced realism’ (cf. Schubert, 2001, and** [**http://www.igroup.org/pq/ipq/index.php**](http://www.igroup.org/pq/ipq/index.php)**)**

**Component 1: Spatial presence**

**Q40.** How aware were you of the real world surroundings while watching the VR concert? (e.g., sounds, room temperature, other people, etc.)

*<7-point Likert scale ranging from ‘extremely aware’ (1) to ‘not aware at all (7)>*

**Q41.** Please indicate which aspects of your real environment continued to draw your attention during the concert. <*free text input>*

**Q42.** Please indicate which aspects of your real environment you no longer paid attention to during the concert. <*free text input>*

**Q43.** Please indicate to what extent you agree or disagree with the following statements regarding your experience of VR concerts.

|  | Strongly disagree (1) | Disagree (2) | Slightly disagree (3) | Neutral (4) | Slightly agree (5) | Agree (6) | Strongly agree (7) |
| --- | --- | --- | --- | --- | --- | --- | --- |
| I felt that the virtual concert space surrounded me. |  |  |  |  |  |  |  |
| I had a sense of acting in the virtual concert space, rather than just perceiving pictures. |  |  |  |  |  |  |  |
| I completely forgot I was in a virtual world and not in a real world. |  |  |  |  |  |  |  |

**Component 2: Involvement**

**Q44.** What was your level of attention during the VR concert?

*<7-point Likert scale ranging from ‘no concentration at all’ (1) to ‘total concentration’ (7)>*

**Q45.** What influenced your level of attention during VR concerts? <*free text input>*

**Q46.** How aware were you of other people in the virtual audience attending the same concert as you?

*<7-point Likert scale ranging from ‘not aware at all’ (1) to ‘completely aware’ (7)>*

**Q47.** Please indicate to what extent you agree or disagree with the following statements regarding your experience of VR concerts.

|  | Strongly disagree (1) | Disagree (2) | Slightly disagree (3) | Neutral (4) | Slightly agree (5) | Agree (6) | Strongly agree (7) |
| --- | --- | --- | --- | --- | --- | --- | --- |
| I felt involved during the VR concert. |  |  |  |  |  |  |  |
| I am interested in VR precisely because it allows experiences that are not possible in the everyday, physical world. |  |  |  |  |  |  |  |

**Component 3: Experienced realism**

**Q48.** Do you think it’s important that VR concerts are realistic? By realistic, we mean the resemblance to a concert you physically attend.

*<7-point Likert scale ranging from ‘not at all’ (1) to ‘absolutely’ (7)>*

**Q49** How real did the VR concert you attended seem to you compared to a concert you physically attend?

*<7-point Likert scale ranging from ‘not real at all’ (1) to ‘completely real’ (7)>*

**Q50.** Please clarify which aspects seemed real to you. <*free text input>*

**Q51.** Please clarify which aspects didn’t seem real to you. <*free text input>*

**CATEGORY 5: Comparison Physical Attendance**

**Q52.** Do you think VR concerts are less or more accessible than concerts you physically attend?

*<7-point Likert scale ranging from ‘far less accessible’ (1) to ‘far more accessible’ (7)>*

**Q53.** What do you think makes VR concerts less or more accessible? <*free text input>*

**Q54.** Do you think concerts in VR are a less or more unique experience than concerts you physically attend?

*<7-point Likert scale ranging from ‘far less unique’ (1) to ‘far more unique’ (7)>*

**Q55.** Please explain why you think VR concerts are less or more unique. <*free text input>*

**Q56.** We have some statements of which we would like to know if you agree with them or not.

|  | Strongly disagree (1) | Disagree (2) | Slightly disagree (3) | Neutral (4) | Slightly agree (5) | Agree (6) | Strongly agree (7) |
| --- | --- | --- | --- | --- | --- | --- | --- |
| The visual spectacle gave the music a new dimension |  |  |  |  |  |  |  |
| I am interested in VR concerts because of the impossible experiences it enhances |  |  |  |  |  |  |  |
| Compared to physical concerts, I could be more myself at VR concerts because no one could see me. |  |  |  |  |  |  |  |
| It’s easier to connect with other people in the audience during a VR concert than a concert you physically attend. |  |  |  |  |  |  |  |
| VR concerts give the same fulfilment as physical concerts. |  |  |  |  |  |  |  |

**Q57.** Compared to concerts where you can be physically present, what do you miss at VR concerts? <*free text input>*

**Q58.** Compared to concerts where you can be physically present, what added value do VR concerts have? <*free text input>*

**CATEGORY 6: Personal View and Future Outlook**

**Q59.** Lastly, we have some statements of which we would like to know if you agree with them or not.

|  | Strongly disagree (1) | Disagree (2) | Slightly disagree (3) | Neutral (4) | Slightly agree (5) | Agree (6) | Strongly agree (7) |
| --- | --- | --- | --- | --- | --- | --- | --- |
| I prefer VR concerts over physical concerts. |  |  |  |  |  |  |  |
| If possible, I would attend more VR concerts. |  |  |  |  |  |  |  |
| VR concerts are the future of the music scene. |  |  |  |  |  |  |  |
| When an artist gives a physical concert as well as a VR concert, I’d still choose the VR concert. |  |  |  |  |  |  |  |

**Q60.** Comments. <*free text input>*
